# A prospective study of serum metabolites and glioma risk

**DOI:** 10.18632/oncotarget.19705

**Published:** 2017-07-31

**Authors:** Jiaqi Huang, Stephanie J. Weinstein, Cari M. Kitahara, Edward D. Karoly, Joshua N. Sampson, Demetrius Albanes

**Affiliations:** ^1^ Metabolic Epidemiology Branch, Division of Cancer Epidemiology and Genetics, National Cancer Institute, NIH, Department of Health and Human Services, Bethesda, MD, USA; ^2^ Radiation Epidemiology Branch, Division of Cancer Epidemiology and Genetics, National Cancer Institute, NIH, Department of Health and Human Services, Bethesda, MD, USA; ^3^ Director of Project Management, Metabolon, Inc., Morrisville, NC, USA; ^4^ Biostatistics Branch, Division of Cancer Epidemiology and Genetics, National Cancer Institute, NIH, Department of Health and Human Services, Bethesda, MD, USA

**Keywords:** malignant glioma, metabolomics, prospective, arginine/proline metabolism, antioxidant

## Abstract

Malignant glioma is one of the most lethal adult cancers, yet its etiology remains largely unknown. We conducted a prospective serum metabolomic analysis of glioma based on 64 cases and 64 matched controls selected from Alpha-Tocopherol, Beta-Carotene Cancer Prevention (ATBC) Study. Median time from collection of baseline fasting serum to diagnosis was nine years (inter-decile range 3-20 years). LC/MS-MS identified 730 known metabolites, and conditional logistic regression models estimated odds ratios for one-standard deviation differences in log-metabolite signals. Forty-three metabolites were associated with glioma at *P*<0.05. 2-Oxoarginine, cysteine, alpha-ketoglutarate, chenodeoxycholate and argininate yielded the strongest metabolite signals and were inversely related to overall glioma risk (0.0065≤*P*<0.0083). Also, seven xanthine metabolites related to caffeine metabolism were higher in cases than in controls (0.017≤*P*<0.042). Findings were mostly similar in high-grade glioma cases, although prominent inversely associated metabolites included the secondary bile acids glycocholenate sulfate and 3β-hydroxy-5-cholenoic acid, xenobiotic methyl 4-hydroxybenzoate sulfate, sex steroid 5alpha-pregnan-3beta, 20beta-diol-monosulfate, and cofactor/vitamin oxalate (0.0091≤*P*<0.021). A serum metabolomic profile of glioma identified years in advance of clinical diagnoses is characterized by altered signals in arginine/proline, antioxidant, and coffee-related metabolites. The observed pattern provides new potential leads regarding the molecular basis relevant to etiologic or sub-clinical biomarkers for glioma.

## INTRODUCTION

Brain cancer is one of the most fatal and devastating malignancies, given its poor prognosis and adverse impact on quality of life, including, particularly, cognitive function. Malignant glioma accounts for 80% of adult brain cancers [1], and its etiology remains largely unknown, with the exception of ionizing radiation and family history, and evidence pointing to inverse associations with asthma and allergies [1–7]. A spectrum of genetic alterations has been characterized for glioma, including germline and somatic mutations, recurrent translocations, and copy number variations, [8, 9] yet these do not account for all the underlying biology. Rapid development of technologies in liquid and gas chromatography, mass spectrometry and nuclear magnetic resonance have facilitated the measurement of a broad array of low molecular weight metabolites in biospecimens such as plasma and serum, urine and tissue. Quantification of the metabolome provides an integrated snapshot reflection of exogenous and endogenous exposures, and may thus help to identify novel disease associations and point to biochemical pathways involved in disease pathogenesis. Chinnaiyan and colleagues demonstrated unique tumor metabolomic signatures, involving cellular energy, anabolism and phospholipid pathways, that distinguished low-grade from high-grade gliomas and had prognostic relevance [10]. The significance of these metabolic differences to the etiology, early detection, and prevention of the disease remains to be established, however, including through prospective investigations [11, 12].

To address the potential role of altered metabolites and their related biological pathways in glioma tumorigenesis, we conducted a prospective case-control serologic analysis including 64 glioma cases nested within the Alpha-Tocopherol, Beta-Carotene Cancer Prevention (ATBC) Study cohort. According to the World Health Organization (WHO) grade for glioma (I-IV) [13], the present study includes 41 high-grade gliomas (grade IV), 19 lower-to-intermediate-grade gliomas (grades II and III; subsequently referred to “lower-grade”), and 4 cases of unknown grade [14].

## RESULTS

Cases were similar to controls with respect to baseline characteristics, with the exception that they had lower average body mass index (BMI) (*P*=0.025) and appeared to consume more coffee (although not statistically significantly different (*P*=0.21) (Table 1). The median time from serum collection to glioma diagnosis was nine years (inter-decile range=3.0-20.0 years). Based on the quality control samples, the median coefficient of variation (CV) across all the metabolites was 7% (interquartile range=4%-14%) and the median intra-class correlation coefficient (ICC) was 0.97 (interquartile range=0.89-0.99).

**Table 1:** Baseline characteristics of glioma cases and controls^1^

	Controls	Cases	*P*-value^2^
***N***	64	64	
**Age at blood draw, years**	58	57	Matched
**Median time from serum collection to diagnosis (years, interdecile range)**	—	9.0 (3.0-20.0)	—
**Height (cm)**	173.9	174.4	0.93
**Weight (kg)**	80.2	77.0	0.069
**BMI (kg/ m**^2^**)**	26.5	25.3	0.025
**History of diabetes (%)**	7.8	3.1	0.24
**Cigarettes per day**	21.1	20.2	0.45
**Years of cigarette smoking**	36.5	35.4	0.57
**Physically active (%)**	18.8	23.4	0.67
**Serum retinol (μg/ L)**	592.3	586.4	0.76
**Serum total cholesterol (mmol/ L)**	6.2	6.2	0.70
**Serum α-tocopherol (mg/ L)**	12.0	11.4	0.057
**Serum β-carotene (μg/ L)**	178.7	214.3	0.55
**Dietary intake per day**			
**Total energy (kcal)**	2776.9	2634.0	0.33
**Fruit (g)**	114.0	135.0	0.25
**Vegetables (g)**	121.2	106.2	0.21
**Red meat (g)**	78.0	67.8	0.059
**Coffee (g)**	558.6	631.6	0.21
**Alcohol (ethanol, g)**	18.0	14.8	0.64
**Supplement use**			
**Vitamin A (%)**	9.4	7.8	1.00
**Vitamin D (%)**	6.3	7.8	1.00
**Calcium (%)**	10.9	12.5	1.00

^1^Values are means unless otherwise indicated. All data were obtained at baseline.

^2^Wilcoxon rank test for continuous variables, or Fisher’s exact test for categorical variables.

Serum metabolites related to risk of overall and high-grade glioma with a nominal *P*-value of <0.05 are listed in Tables 2 and 3, respectively, sorted by chemical class (also known as “super-pathway”), sub-class (also known as “sub-pathway”) and *P-*values. No association reached significance after Bonferroni correction for multiple comparisons (threshold of *P*=0.000068). In the primary analysis of all cases and controls, we found that the amino acids 2-oxoarginine, cysteine and argininate, energy metabolite alpha-ketoglutarate, and lipid chenodeoxycholate yielded the strongest signals, being lower in cases than in controls (0.0065≤P<0.0083, Table 2). Amino acid metabolites in the phenylalanine/tyrosine sub-pathway (N-acetyltyrosine, N-acetylphenylalanine, phenyllactate and tyrosine; 0.011≤*P*<0.048), and the tryptophan sub-pathway (N-acetylkynurenine, N-acetyltryptophan and xanthurenate; 0.042≤*P*<0.045) were also lower in cases. By contrast, the serum acylcarnitines stearoylcarnitine, margaroylcarnitine, and eicosenoylcarnitine (0.016≤*P*<0.046), and xanthine (i.e., caffeine) metabolites 1-methylurate, 1-methylxanthine, paraxanthine, theobromine, 5-acetylamino-6-amino-3-methyluracil, theophylline, and 7-methylxanthine (0.017≤*P*<0.042) were higher in cases than in controls (Table 2), as were serum levels of 2-hydroxyglutarate (2-HG) (odds ratio (OR)=1.53, 95% confidence interval (CI): 0.98, 2.39, *P*=0.06; data not shown). Adjustment for age at baseline or diagnosis, BMI, history of diabetes, height, physical activity, cigarettes per day, or red meat or alcohol consumption did not alter the findings (data not shown).

**Table 2:** Serum metabolites related to risk of overall glioma (*P* < 0.05)^1^

Metabolite	Sub-pathway	Odds Ratio	95% CI	*P* value	*P* for chemical class
**Amino acids and amino acid derivatives**					0.14
Glutamate	Glutamate metabolism	0.65	0.43, 0.96	0.0321	
N-Acetylleucine	Leucine, isoleucine and valine metabolism	0.67	0.44, 1.00	0.0499	
Cysteine	Methionine, cysteine, SAM and taurine metabolism	0.39	0.19, 0.77	0.0069	
Cysteine-S-sulfate	Methionine, cysteine, SAM and taurine metabolism	0.62	0.40, 0.96	0.0323	
N-Acetyltyrosine	Phenylalanine and tyrosine metabolism	0.57	0.36, 0.88	0.0109	
N-Acetylphenylalanine	Phenylalanine and tyrosine metabolism	0.63	0.41, 0.96	0.0326	
Phenyllactate (PLA)	Phenylalanine and tyrosine metabolism	0.67	0.46, 0.97	0.0350	
Tyrosine	Phenylalanine and tyrosine metabolism	0.71	0.50, 1.00	0.0477	
N-Acetylkynurenine	Tryptophan metabolism	0.65	0.43, 0.98	0.0420	
N-Acetyltryptophan	Tryptophan metabolism	0.64	0.41, 0.98	0.0421	
Xanthurenate	Tryptophan metabolism	0.67	0.45, 0.99	0.0453	
2-Oxoarginine	Urea cycle; arginine and proline metabolism	0.56	0.37, 0.85	0.0065	
Argininate	Urea cycle; arginine and proline metabolism	0.60	0.42, 0.88	0.0083	
N-Acetylarginine	Urea cycle; arginine and proline metabolism	0.69	0.48, 0.98	0.0407	
**Carbohydrates**					0.36
Mannitol/sorbitol	Fructose, mannose and galactose metabolism	0.63	0.41, 0.97	0.0374	
Pyruvate	Glycolysis, gluconeogenesis, and pyruvate metabolism	0.65	0.43, 0.97	0.0343	
**Cofactors and Vitamins**					0.23
Trigonelline (N'-methylnicotinate)	Nicotinate and nicotinamide metabolism	1.72	1.10, 2.69	0.0182	
Alpha-tocopherol	Tocopherol metabolism	0.65	0.44, 0.96	0.0305	
**Energy**					0.25
Alpha-ketoglutarate	TCA cycle	0.52	0.32, 0.84	0.0075	
**Lipids**					0.36
Stearoylcarnitine (C18)	Fatty acid metabolism (Acyl carnitine)	1.58	1.09, 2.29	0.0159	
Margaroylcarnitine	Fatty acid metabolism (Acyl carnitine)	1.50	1.05, 2.15	0.0251	
Eicosenoylcarnitine (C20:1)	Fatty acid metabolism (Acyl carnitine)	1.59	1.01, 2.51	0.0457	
1-Palmitoyl-2-linoleoyl-GPI (16:0/18:2)	Phospholipid metabolism	0.61	0.41, 0.91	0.0147	
Glycerophosphorylcholine (GPC)	Phospholipid metabolism	1.76	1.00, 3.10	0.0484	
1-(1-Enyl-palmitoyl)-2-oleoyl-GPC (P-16:0/18:1)	Plasmalogen	1.47	1.01, 2.15	0.0449	
Chenodeoxycholate	Primary bile acid metabolism	0.56	0.37, 0.86	0.0082	
Cholate	Primary bile acid metabolism	0.6	0.39, 0.91	0.0162	
3β-Hydroxy-5-cholenoic acid	Secondary bile acid metabolism	0.67	0.45, 0.98	0.0393	
Glycocholenate sulfate	Secondary bile acid metabolism	0.64	0.41, 0.98	0.0420	
Sphingomyelin (d18:1/17:0, d17:1/18:0, d19:1/16:0)	Sphingolipid metabolism	1.67	1.07, 2.61	0.0228	
**Nucleotides**					0.59
Cytidine	Pyrimidine metabolism, cytidine containing	1.49	1.01, 2.19	0.0471	
**Peptides**					0.64
Gamma-glutamyltyrosine	Gamma-glutamyl amino acid	0.61	0.40, 0.92	0.0175	
**Xenobiotics**					0.58
Propyl 4-hydroxybenzoate sulfate	Benzoate metabolism	0.54	0.32, 0.92	0.0230	
Methyl 4-hydroxybenzoate sulfate	Benzoate metabolism	0.67	0.46, 0.97	0.0320	
3-Methyl catechol sulfate	Benzoate metabolism	1.53	1.02, 2.28	0.0380	
Quinate	Food component/plant	1.52	1.03, 2.25	0.0334	
1-Methylurate	Xanthine metabolism	1.58	1.08, 2.30	0.0171	
1-Methylxanthine	Xanthine metabolism	1.63	1.09, 2.46	0.0184	
Paraxanthine	Xanthine metabolism	1.52	1.05, 2.22	0.0284	
Theobromine	Xanthine metabolism	1.53	1.02, 2.28	0.0375	
5-Acetylamino-6-amino-3-methyluracil	Xanthine metabolism	1.55	1.02, 2.35	0.0379	
Theophylline	Xanthine metabolism	1.50	1.02, 2.22	0.0412	
7-Methylxanthine	Xanthine metabolism	1.47	1.01, 2.14	0.0415	

^1^Conditional logistic regression models were used to estimate odds ratio and their 95% confidence intervals (CIs), with only matching factors adjusted in the model. The odds ratio is for one-standard deviation increase in metabolite level. The table is sorted by chemical class, sub-pathway, and *P* value. The analysis is based on 64 cases and 64 controls.

**Table 3:** Serum metabolites related to risk of high-grade glioma (*P* < 0.05)^1^

Metabolite	Sub-pathway	Odds Ratio	95% CI	*P* value	*P* for chemical class
**Amino acids and amino acid derivatives**					0.85
N-Acetylglutamate	Glutamate metabolism	1.99	1.10, 3.61	0.0228	
2,3-Dihydroxy-2-methylbutyrate	Leucine, isoleucine and valine metabolism	2.05	1.09, 3.85	0.0258	
Cysteine	Methionine, cysteine, SAM and taurine metabolism	0.44	0.20, 0.98	0.0437	
**Carbohydrates**					0.48
Ribonate	Pentose metabolism	2.53	1.02, 6.25	0.0445	
**Cofactors and Vitamins**					0.07
Oxalate (ethanedioate)	Ascorbate and aldarate metabolism	0.55	0.33, 0.91	0.0211	
Threonate	Ascorbate and aldarate metabolism	0.57	0.34, 0.95	0.0300	
Gulonate	Ascorbate and aldarate metabolism	1.99	1.04, 3.81	0.0387	
**Energy**					0.49
Aconitate [cis or trans]	TCA cycle	1.70	1.00, 2.89	0.0502	
**Lipids**					0.97
Oleoyl-oleoyl-glycerol (18:1/18:1)	Diacylglycerol	1.85	1.03, 3.31	0.0402	
Cholate	Primary bile acid metabolism	0.54	0.31, 0.93	0.0273	
Glycocholenate sulfate	Secondary bile acid metabolism	0.39	0.19, 0.79	0.0091	
3β-Hydroxy-5-cholenoic acid	Secondary bile acid metabolism	0.49	0.28, 0.85	0.0106	
5Alpha-pregnan-3beta, 20beta-diol monosulfate	Steroid	0.56	0.34, 0.92	0.0209	
Pregnenolone sulfate	Steroid	0.53	0.30, 0.94	0.0293	
**Nucleotides**					0.15
5-Methyluridine (ribothymidine)	Pyrimidine metabolism, uracil containing	2.25	1.12, 4.52	0.0226	
**Xenobiotics**					0.63
Tartronate (hydroxymalonate)	Bacterial/fungal	0.57	0.34, 0.97	0.0383	
Methyl 4-hydroxybenzoate sulfate	Benzoate metabolism	0.47	0.26, 0.83	0.0097	
Propyl 4-hydroxybenzoate sulfate	Benzoate metabolism	0.52	0.28, 0.97	0.0391	

^1^Conditional logistic regression models were used to estimate odds ratio and their 95% confidence intervals (CIs), with only matching factors adjusted in the model. The odds ratio is for one-standard deviation increase in metabolite level. The table is sorted by chemical class, sub-pathway, and *p* value. The grade is based on World Health Organization Grade of glioma, and Grade IV is defined as high-grade. The analysis is based on 41 cases and 41 controls.

High-grade glioma showed fewer but similar risk associations overall, although proportionally more metabolites were elevated in cases relative to controls (e.g., N-acetylglutamate, ribonate, and 5-methyluridine; 0.04≤*P*<0.02; Table 3). Notably, xanthine compounds appeared unrelated to high-grade glioma. Prominent metabolite signals with inverse risk associations were the secondary bile acids glycocholenate sulfate and 3β-hydroxy-5-cholenoic acid, xenobiotic methyl 4-hydroxybenzoate sulfate, sex steroid 5alpha-pregnan-3beta, 20beta-diol monosulfate, and cofactor/vitamin oxalate (ethanedioate) (0.0091≤*P*<0.021; Table 3).

Stratifying cases and their controls by median time from blood draw to diagnosis showed that several lysoplasmalogen, sphingolipid, and three of four benzoate metabolites were positively related to glioma within nine years of blood collection (Supplementary Table 1). By contrast, abundant diacylglycerol, monoacylglycerol, phospholipid, and sphingolipid metabolites were prominently and inversely related to cases that were diagnosed at least nine years after blood collection (Supplementary Table 2). We found that caffeine intake did not modify the association between identified caffeine related metabolites and glioma risk (Supplementary Table 3).

None of the top 10 principal components were significantly associated with glioma risk, with all tests showing *P*≥0.034 (*P*<0.005 being the threshold). The Gene-Set Analysis (GSA) pathway analysis revealed primary bile acid, urea cycle/arginine and proline, tocopherol, and glycolysis/gluconeogenesis/pyruvate associations with overall glioma risk (0.005≤*P*<0.048; Table 4). Ascorbate and aldarate metabolites appeared to be related to high-grade glioma (*P*=0.02), while glutamate, glycolysis/gluconeogenesis/pyruvate (i.e., cellular energy), eicosanoid and alanine/aspartate metabolites were related to lower-grade glioma (*P*=0.006, 0.017, 0.03, 0.04, respectively; Table 4). The sub-pathway analysis stratified by time from blood collection to diagnosis revealed that primary bile acid metabolites were related to cases diagnosed within nine years of blood collection (*P*=0.034), whereas urea cycle/arginine and proline, glycolysis/gluconeogenesis/pyruvate, monoacylglycerol, histidine, food component/plant, and diacylglycerol metabolites were related to risk of glioma after nine years (0.003≤*P*<0.045, Supplementary Table 4). These pathway associations did not, however, reach the stringent Bonferroni significance threshold for correction of multiple comparisons [i.e., eight tests for super-pathways (*P*=0.0063) and 70 tests for sub-pathways (*P*=0.00071)].

**Table 4:** Gene set analysis (GSA) for sub-pathways of serum metabolites and risk of glioma (*P* < 0.05)^1^

Sub-pathway	No. of contributing metabolites	*P* value from GSA
**Overall glioma**		
Primary bile acid metabolism	8	0.005
Urea cycle; arginine and proline metabolism	16	0.032
Tocopherol metabolism	3	0.034
Fatty acid, amino	2	0.045
Glycolysis, gluconeogenesis, and pyruvate metabolism	5	0.048
**High-grade glioma**		
Ascorbate and aldarate metabolism	3	0.02
**Lower-grade glioma**		
Glutamate metabolism	9	0.006
Glycolysis, gluconeogenesis, and pyruvate metabolism	5	0.017
Eicosanoid	2	0.03
Alanine and aspartate metabolism	6	0.04

^1^GSA, a standard pathway method, was used to examine whether the pre-defined sub-pathways were associated with glioma status. The grade is based on the World Health Organization Grade of glioma, and Grade IV is defined as high-grade, and Grade II-III is defined as lower-grade. The GSA sub-pathway analysis for overall glioma is based on 64 cases and 64 controls, for high-grade glioma is based on 41 cases and 41 controls, for lower-grade glioma is based on 19 cases and 19 controls.

## DISCUSSION

Our study identified the amino acids 2-oxoarginine, cysteine and argininate, energy metabolite alpha-ketoglutarate, and secondary bile acid chenodeoxycholate, as well as some other compounds, as being lower in circulation of glioma cases years in advance of diagnosis compared to study-based controls. By contrast, we show several xanthine metabolites of caffeine to be related to higher risk. Ascorbate and aldarate metabolites are associated with high-grade glioma, while glycolysis/gluconeogenesis/pyruvate, eicosanoid and glutamate metabolites appeared related to lower-grade disease.

A possible role for coffee consumption in the etiology of glioma has long been hypothesized, and a meta-analysis of four prospective and two retrospective studies showed no association [15]. Previous research has identified several coffee-related metabolites in circulation, including quinate, 1-methylurate, 1-methylxanthine, paraxanthine, theobromine, 5-acetylamino-6-amino-3-methyluracil, theophylline, 7-methylxanthine, and trigonelline [16], with many of these compounds showing substantial positive associations with overall glioma risk in the present investigation. Whether these xanthine metabolites have a direct causal role in glioma, or are elevated years in advance of diagnosis because of increased coffee consumption in response to tumor-related neurologic changes or cancer-related fatigue, for example, will require additional clinical and prospective studies.

The arginine/proline metabolic pathway compounds 2-oxoarginine, a guanidino metabolite of arginine, and argininate, the conjugate base of arginine, were substantially lower in men years in advance - and especially nine or more years - of being diagnosed with glioma compared with non-cases. A recent plasma metabolomic analysis of 87 glioma patients found lower arginine concentrations in high-grade glioma compared to low-grade, suggesting greater arginine dependence of the former [17]. Earlier studies have indicated that arginine/proline metabolites are involved in tumorigenesis (including glioblastoma), exogenous arginine is required for tumor growth, and arginine deprivation leads to impairment of glioma cell motility, invasiveness, and adhesion [18–21].

A large proportion (i.e., 60-90%) of low-grade gliomas harbor a heterozygous mutation (R132H) in the gene encoding the cytosolic isoform of isocitrate dehydrogenase (IDH1) [22]. Data from genome-wide association studies of glioma have identified single nucleotide polymorphisms (SNP) associated with altered risk of *IDH*-mutated glioma, including rs55705857 in 8q24.21, rs4295627 in *CCDC26*, and rs498872 in *PHLDB1* [23, 24], and in a recent case-control study of 285 gliomas, 316 healthy controls, and 531 other types of cancers, the authors showed that SNP rs55705857 was strongly associated with altered risk of *IDH*-mutant glioma, but not with other cancers [23]. The wild-type IDH1 catalyzes the oxidative decarboxylation of isocitrate to generate alpha-ketoglutarate (α-KG), whereas the mutant enzyme is able to convert α-KG into molecules of 2-HG, which is an “oncometabolite” that may mediate several tumorigenic events [25–27]. In the present study, we observed increased pre-diagnostic serum 2-HG and decreased α-KG in glioma cases. We could not, however, evaluate *IDH1* mutation status and its correlation with serum 2-HG, although previous studies indicated no correlation between serum 2-HG and *IDH1/2* status or tumor size [17, 28]. On the other hand, it is unlikely that the serum 2-HG and α-KG reflect early cellular changes in transformed astrocytes or emerging gliomas, but rather etiologic biomarkers that must be considered and further evaluated in other studies. It is of note that we observed that another TCA cycle metabolite, aconitate (related to cis-aconitate), was elevated in high-grade cases (OR=1.7, *P*=0.05).

Being a highly metabolically active organ, the brain generates substantial reactive oxygen species (ROS) and is slower to neutralize these free radicals compared to other tissues [29], possibly leading to DNA damage, genomic instability and tumor development. We found several antioxidant pathway metabolites inversely associated with risk that may be indicative of serum antioxidant depletion resulting from increased tumor ROS. For example, both cysteine and cysteine-S-sulfate were lower in cases, and this might be associated with increased cysteine uptake, and modulating of redox status, in the central nervous system. The conditionally essential amino acid cysteine is a rate-limiting precursor for the antioxidant glutathione, one of the most abundant antioxidants in the central nervous system [30, 31]. Whereas cysteine is an extracellular antioxidant, glutathione acts intracellularly and may play a role in glioma cell survival under redox stress and hypoxic conditions [30–32]. Consistent with this is a metabolomic study of patient-derived glioma tissue that found cysteine catabolism and cysteine sulfinic acid accumulation in the high-grade glioblastoma cases [33]. Higher circulating cysteine has also been related to lower risks of several other cancers including colon [34], esophagus, and stomach [35].

Also relevant to ROS and glioma risk is the inverse association we observed for alpha-tocopherol, the most biologically active form of vitamin E and a potent inhibitor of lipid peroxidation [36]. Two previous studies had similar findings for glioma and glioblastoma [37, 38], while a recent report showed positive associations for several antioxidants including alpha-tocopherol [39]. The latter finding was, however, restricted to risk 10-22 years after blood collection [39]. By contrast, and also relevant to the metabolite-ROS-glioma associations, we showed two of three compounds in the ascorbate/aldarate pathway were inversely associated with high-grade disease.

5Alpha-pregnan-3beta, 20beta-diol monosulfate and pregnenolone sulfate were reduced in high-grade glioma cases. The latter is considered a neurosteroid that can be synthesized in the central nervous system, is present in higher concentrations in brain tissue than in plasma [40, 41], and is a precursor of some neurosteroids characterized as having neuroprotective effects [42, 43]. Experimental data also indicate that pregnenolone can regulate glioma cell death through extrinsic and intrinsic apoptotic pathways in a caspase-dependent manner [44].

Altered energy metabolism has been considered one of the hallmarks of cancer [45]. Glycolysis, as a highly conserved metabolic process, is essential for energy production in normal mammalian cells, and impairment of glycolysis has been depicted as a feature of cancer metabolism; i.e., the Warburg effect [46–48]. Impairment of glycolysis in glioma has been found in the present study (especially in low-grade tumors), as well as in previous studies (distinguishing low- and high-grade tumors) [10, 17]. One of the previous studies [10] that identified a total of 308 known biochemicals and used fresh-frozen tumor tissue found alterations in glucose metabolism as a function of tumor grade, especially distinguishing grade IV tumors from grade II (33 grade IV tumors and 18 grade II tumors). Another study [17] using serum that detected a total of 224 known metabolites from 25 key metabolic pathways reported that the carbohydrate pathway (e.g. glycolysis or gluconeogenesis) was ranked 6th of 18 metabolic pathways that significantly differed between high- and low-grade glioma. They have defined low-grade glioma (N=42) as grade I and II, and high-grade (N=45) glioma as grade III and IV. Based on differences in study designs, including biospecimen type (serum *vs*. tissue), reference groups (healthly controls in the present study *vs*. low-grade glioma cases in previous clinical studies), number of identified metabolites (730 known metabolites in the present study *vs*. 200-300 metabolites in the previous studies), and definition of low- and high-grade glioma (grade II and III as low-grade and grade IV as high-grade in the present study *vs*. grade I/II as low-grade and grade III/IV as high grade), a direct comparison across the studies is not facile and involves imprecision. Our findings suggest that impairment of the glycolysis pathway may be an early event during glioma development, likely to support cell proliferation and tumor anabolic activity. Of note, impairment of glycolysis has been shown in earlier studies to be associated with activated oncogenes (such as *RAS*, or *MYC*) and mutant tumor suppressors (such as TP53) [45, 49, 50] and such mutations have been reported to occur more frequently in low-grade rather than high-grade gliomas [51–53].

Notable strengths of our investigation include assaying of overnight fasting serum samples that were obtained up to two decades prior to the diagnosis of glioma. Cases were ascertained from census-based population registers with high accuracy. Untargeted metabolomic profiling was able to identify several hundred metabolites using a high-quality platform with careful quality control and laboratory blinding to case-control status. Study limitations include the homogenous nature of the Finnish male smoker population which impacts the generalizability of our findings to other populations. Also, serum metabolites were only measured at one blood sampling time-point, whereas additional time-points would have provided a more robust reflection of one’s usual or average profile. We are unable to access tumor tissue for glioma genotyping, such as for *IDH* mutation, thus we could not classify gliomas based on this factor. Our study sample size was relatively small for an agnostic investigation, albeit glioma is a relatively rare malignancy and all available cases were studied. Although none of our findings exceeded statistically significant thresholds for multiple comparisons, a large number of the metabolites were highly correlated with one another (e.g., fatty acids), which makes the Bonferroni threshold particularly stringent in that the tests were not completely independent. Nonetheless, our findings should be considered preliminary and hypothesis-generating, even with respect to top signals that were consistent with data from previous studies.

In conclusion, the present study finds a serum metabolomic profile of glioma up to 20 years prior to clinical diagnosis that is characterized by altered molecular signals in arginine/proline, antioxidant, and coffee-related metabolites. Ascorbate/aldarate and steroid hormone metabolites were found to be associated with high-grade glioma. The observed profiles provide evidence regarding the molecular basis relevant to etiologic or sub-clinical biomarkers for glioma. Further prospective metabolomic studies are needed to re-examine the findings in larger and more diverse populations.

## MATERIALS AND METHODS

### Study population

The ATBC Study was a 2×2 factorial, randomized, double-blind, placebo-controlled primary prevention trial originally conducted to examine whether α-tocopherol and β-carotene supplementation could reduce incidence of cancer [54]. Details of the study have been described [54]. Briefly, the trial enrolled Caucasian male smokers (n=29,133), aged 50-69 years from 1985 to 1988 in southwest Finland. The participants were randomly assigned to receive one of four supplements: α-tocopherol (50 mg/day), β-carotene (20 mg/day), both or placebo for 5-8 years (median=6.1) through the end of the trial (April 30, 1993). Pre-supplementation fasting blood samples from all participants (years prior to cancer diagnoses) were collected during the baseline visit, and stored at -70 °C until assessment. At enrollment, self-reported questionnaires were completed with information regarding general health, behavioral and lifestyle factors, and height and weight were measured. Participants were followed from date of enrollment, to date of glioma diagnosis (Finnish Cancer Registry), date of death (Finnish Register of Causes of Death), or censor date (December 31, 2012), whichever occurred first.

All participants provided written informed consent at enrollment. The ATBC Study was approved by institutional review boards at the U.S. National Cancer Institute and the Finnish National Institute for Health and Welfare.

### Case ascertainment and control selection

A total of 64 glioma cases (ICD-9 191, ICD-O morphology code 9380-9481), including 41 high-grade, 19 lower-grade and 4 unknown grade, ascertained during the follow-up period are included in the present analysis. Using incidence-density sampling without replacement, we randomly selected 64 matched controls based on age (± 1 year) and date of blood collection (± 30 days).

### Metabolite assessment

Ultrahigh performance liquid chromatograph/tandem mass spectrometry (LC-MS/MS), a high resolution accurate mass (HRAM) platform at Metabolon Inc., was used to determine serum metabolomic profiles as previously described [55, 56]. To summarize, extraction of samples was processed using an automated liquid handling robot (Hamilton LabStar, Hamilton Robotics, Inc., Reno, NV), and 450μl of methanol was added to 100 μl of sample to precipitate proteins. To confirm extraction efficiency, four recovery standards were added to the methanol, including DL-2-fluorophenylglycine, tridecanoic acid, d6-cholesterol and 4-chlorophenylalanine. Four aliquots from each sample were obtained and dried. For the negative ion analysis, two aliquots of each serum sample were reconstituted in 50μl of 6.5 mM ammonium bicarbonate in water with a pH of 8. For the positive ion analysis, another two aliquots of each serum sample were reconstituted using 50μl 0.1% formic acid in water with a pH of 3.5. The procedures further included raw data extraction, peak-identification and quality control (QC) inclusion in each assay. The assays were run in four batches, each batch contained 16 case-control pairs and two QC samples. We identified a total of 1,064 metabolites, of which 311 metabolites were unknown, and 753 were known molecules. We excluded 23 metabolites that were missing (i.e. below the limit of detection) in >110 study individuals (86%), leaving a total of 730 identified compounds in the final analysis. Metabolites were categorized into one of eight mutually exclusive chemical classes: amino acids and amino acid derivatives (subsequently refer to as “amino acids”), carbohydrates, cofactors and vitamins, energy metabolites, lipids, nucleotides, peptides or xenobiotics. CVs and ICCs were used to assess the data reliability. The CV is defined as the square root of the within-subject variance divided by the mean value. The lower the CV, the better the assay repeatability. The ICC is defined as the between-individual variance divided by the total variance. The range of ICC values are 0-1, and close to 0 suggests little to null reproducibility, whereas close to 1 indicates good reproducibility. Rosner has proposed the classification of ICCs as poor (<0.4), fair-to-good (0.4-0.75), and excellent (≥0.75) [57].

### Statistical analyses

We used either Wilcoxon rank sum (for continuous variables) or Fisher’s exact test (for categorical variables) to compare the demographic characteristics of cases and controls. To standardize the batch variability, we normalized each metabolite within a given batch by dividing by the batch mean of all non-missing values. Metabolites with missing values were imputed to the minimum of the observed values. Metabolite levels were then log-transformed and normalized to have mean = 0 and variance = 1. We modeled the association between glioma and each normalized log-transformed metabolite level by conditional logistic regression and report the ORs for a 1-standard deviation (SD) increase and their 95% CIs, with only matching factors adjusted in the final model. The threshold for statistical significance was defined by Bonferroni correction in the primary analysis with 730 tests (*P*=0.000068). We next performed principle component analysis [58] and repeated the conditional logistic regression for each of the top 10 principal components. We then used GSA, a standard pathway method, to examine whether any of the pre-defined sub or super-pathways were associated with glioma status [59].

Sensitivity analyses were performed, including restriction to high-grade case-control pairs, and stratification by age at enrollment (<56 *vs*. ≥56 years, median age as cutoff point), time to diagnosis (<9 *vs*. ≥9 years, median time as cutoff point), and caffeine intake (<560 *vs*. ≥560 gram, median as cutoff point). Additional analyses adjusted (separately) for BMI (continuous), height (continuous), history of diabetes (yes or no), physical activity (no activity *vs*. at least light activity), and number of daily cigarettes, red meat consumption, and alcohol consumption (all continuous).

The R statistical language version 3.2.3 (Vienna, Austria) was used for GSA analysis, and SAS software version 9.3 (SAS Institute, Cary, NC) was used for other analyses. All presented *P*-values are two-sided.

## SUPPLEMENTARY MATERIALS TABLES


