# Supplementary material for: A prospective study of serum metabolites and glioma risk
Source: Oncotarget. 2017 Jul 31;8(41):70366–77. doi: 10.18632/oncotarget.19705 (PMC5642561; doi:10.18632/oncotarget.19705)
Supplement: Supplementary file 1 [file oncotarget-08-70366-s001.pdf]

A prospective study of serum metabolites and glioma risk

SUPPLEMENTARY MATERIALS

Supplementary Table 1: Serum metabolites related to risk of glioma: 0-9 years from blood draw to glioma diagnosis (*P* < 0.05)[1]

Metabolite	Sub-pathway	Odds Ratio	95% CI	*P value*	*P* for Chemical Class
**Amino Acids and Amino Acid Derivatives**					0.39
Leucine	Leucine, isoleucine and valine metabolism	0.52	0.28, 0.96	0.0378	
2-Hydroxy-3-methylvalerate	Leucine, isoleucine and valine metabolism	0.51	0.27, 0.97	0.0415	
2,3-Dihydroxy-2-methylbutyrate	Leucine, isoleucine and valine metabolism	1.82	1.01, 3.31	0.0479	
Phenyllactate (PLA)	Phenylalanine and tyrosine metabolism	0.51	0.27, 0.95	0.0335	
O-Cresol sulfate	Phenylalanine and tyrosine metabolism	2.20	1.06, 4.56	0.0350	
**Lipids**					0.72
1-(1-Enyl-palmitoyl)-GPC (P-16:0)	Lysoplasmalogen	1.98	1.02, 3.86	0.0436	
1-(1-Enyl-stearoyl)-GPE (P-18:0)	Lysoplasmalogen	1.95	1.02, 3.72	0.0437	
1-Palmitoyl-2-linoleoyl-GPI (16:0/18:2)	Phospholipid metabolism	0.44	0.22, 0.87	0.0181	
Eicosapentaenoylcholine	Phospholipid metabolism	1.92	1.01, 3.68	0.0476	
Cholate	Primary bile acid metabolism	0.42	0.19, 0.93	0.0328	
Glycochenodeoxycholate	Primary bile acid metabolism	0.52	0.29, 0.96	0.0352	
Chenodeoxycholate	Primary bile acid metabolism	0.52	0.27, 1.00	0.0495	
Glycodeoxycholate	Secondary bile acid metabolism	0.50	0.25, 1.00	0.0484	
Sphingomyelin (d18:2/24:1, d18:1/24:2)	Sphingolipid metabolism	2.76	1.24, 6.16	0.0130	
Sphingomyelin (d18:1/18:1, d18:2/18:0)	Sphingolipid metabolism	2.85	1.20, 6.76	0.0178	
Sphingomyelin (d18:1/17:0, d17:1/18:0, d19:1/16:0)	Sphingolipid metabolism	2.20	1.09, 4.46	0.0282	
Palmitoyl sphingomyelin (d18:1/16:0)	Sphingolipid metabolism	2.07	1.05, 4.05	0.0344	
Sphingomyelin (d18:2/23:1)	Sphingolipid metabolism	2.16	1.00, 4.67	0.0488	
**Xenobiotics**					0.22
3-Methyl catechol sulfate	Benzoate metabolism	2.26	1.11, 4.58	0.0237	
Methyl 4-hydroxybenzoate sulfate	Benzoate metabolism	0.49	0.26, 0.92	0.0258	
2-Ethylphenylsulfate	Benzoate metabolism	3.01	1.11, 8.17	0.0300	
3-Methyl catechol sulfate	Benzoate metabolism	2.06	1.06, 3.99	0.0323	
3-Hydroxypyridine sulfate	Chemical	2.30	1.04, 5.12	0.0408	
Isoeugenol sulfate	Food component/plant	2.10	1.09, 4.03	0.0256	
4-Vinylguaiacol sulfate	Food component/plant	2.51	1.04, 6.06	0.0414	
N-(2-Furoyl)glycine	Food component/plant	2.50	1.02, 6.14	0.0455	

[1]Conditional logistic regression models were used to estimate odds ratio and their 95% confidence intervals (CIs), with only matching factors adjusted in the model. The odds ratio is for one-standard deviation increase in metabolite level. The table is sorted by chemical class, sub-pathway, and *P* value. The analysis is based on 31 cases and 31 controls.

Supplementary Table 2: Serum metabolites related to risk of glioma: ≥9 years from blood draw to glioma diagnosis ($P < 0.05$)[1]

Metabolite	Sub-pathway	Odds Ratio	95% CI	P value	P for Chemical Class
**Amino Acids and Amino Acid Derivatives**					0.19
Creatine	Creatine metabolism	0.45	0.23, 0.89	0.0218	
4-Hydroxyglutamate	Glutamate metabolism	0.39	0.19, 0.80	0.0103	
Pyroglutamine	Glutamate metabolism	2.14	1.16, 3.94	0.0147	
Betaine	Glycine, serine and threonine metabolism	1.90	1.02, 3.52	0.0425	
N-Acetyl-1-methylhistidine	Histidine metabolism	0.55	0.33, 0.94	0.0287	
1-Methylimidazoleacetate	Histidine metabolism	0.40	0.17, 0.95	0.0376	
Cysteine S-sulfate	Methionine, cysteine, SAM and taurine metabolism	0.38	0.16, 0.90	0.0271	
Dopamine 3-O-sulfate	Phenylalanine and tyrosine metabolism	0.27	0.11, 0.67	0.0046	
N-Acetyltyrosine	Phenylalanine and tyrosine metabolism	0.51	0.29, 0.90	0.0209	
Tyramine O-sulfate	Phenylalanine and tyrosine metabolism	0.47	0.23, 0.98	0.0436	
N-Acetyltryptophan	Tryptophan metabolism	0.38	0.16, 0.89	0.0263	
N-Acetylkynurenine	Tryptophan metabolism	0.52	0.27, 0.99	0.0464	
Argininate	Urea cycle; arginine and proline metabolism	0.31	0.14, 0.68	0.0036	
2-Oxoarginine	Urea cycle; arginine and proline metabolism	0.23	0.08, 0.64	0.0048	
Ornithine	Urea cycle; arginine and proline metabolism	2.27	1.14, 4.53	0.0204	
Pro-hydroxy-pro	Urea cycle; arginine and proline metabolism	1.86	1.01, 3.41	0.0466	
**Carbohydrates**					0.14
Mannitol/sorbitol	Fructose, mannose and galactose metabolism	0.34	0.12, 0.92	0.0333	
Pyruvate	Glycolysis, gluconeogenesis, and pyruvate metabolism	0.50	0.27, 0.91	0.0238	
**Cofactors and Vitamins**					0.08
Trigonelline (N’-methylnicotinate)	Nicotinate and nicotinamide metabolism	1.86	1.02, 3.37	0.0427	
N1-Methyl-4-pyridone-3-carboxamide	Nicotinate and nicotinamide metabolism	0.49	0.24, 1.00	0.0499	
Pantothenate	Pantothenate and CoA metabolism	0.50	0.26, 0.96	0.0389	
**Energy**					0.28
Succinylcarnitine	TCA cycle	0.24	0.09, 0.69	0.0075	
Alpha-ketoglutarate	TCA cycle	0.52	0.27, 1.00	0.0498	
**Lipids**					0.40
Palmitoleoyl-arachidonoyl-glycerol (16:1/20:4)	Diacylglycerol	0.40	0.18, 0.86	0.0196	
Linoleoyl-docosahexaenoyl-glycerol (18:2/22:6)	Diacylglycerol	0.56	0.33, 0.93	0.0255	
Oleoyl-arachidonoyl-glycerol (18:1/20:4)	Diacylglycerol	0.55	0.31, 0.96	0.0364	
Stearoyl-arachidonoyl-glycerol (18:0/20:4)	Diacylglycerol	0.50	0.26, 0.97	0.0405	
Stearoyl-arachidonoyl-glycerol (18:0/20:4)	Diacylglycerol	0.54	0.30, 0.97	0.0407	
Linoleoyl-arachidonoyl-glycerol (18:2/20:4)	Diacylglycerol	0.58	0.34, 0.99	0.0458	
Margaroylcarnitine	Fatty Acid metabolism (Acyl carnitine)	1.92	1.09, 3.37	0.0233	
Eicosenoylcarnitine (C20:1)	Fatty Acid metabolism (Acyl carnitine)	2.84	1.15, 6.99	0.0234	
Myristoylcarnitine (C14)	Fatty Acid metabolism (Acyl carnitine)	1.92	1.00, 3.66	0.0489	
3-Methylglutarate/2-methylglutarate	Fatty acid, dicarboxylate	3.14	1.35, 7.35	0.0082	
Adipate	Fatty acid, dicarboxylate	2.13	1.10, 4.12	0.0251	
Sebacate (decanedioate)	Fatty acid, dicarboxylate	2.30	1.02, 5.17	0.0439	
3-Hydroxysebacate	Fatty acid, monohydroxy	2.38	1.05, 5.38	0.0369	
1-Dihomo-linolenylglycerol (20:3)	Monoacylglycerol	0.33	0.14, 0.77	0.0106	
1-Docosahexaenoylglycerol (22:6)	Monoacylglycerol	0.44	0.21, 0.91	0.0265	
1-Linolenoylglycerol (18:3)	Monoacylglycerol	0.53	0.29, 0.98	0.0442	
1-Linoleoylglycerol (18:2)	Monoacylglycerol	0.59	0.36, 0.99	0.0456	
1-Stearoyl-2-arachidonoyl-GPC (18:0/20:4)	Phospholipid metabolism	0.50	0.28, 0.89	0.0195	
1-Stearoyl-2-arachidonoyl-GPI (18:0/20:4)	Phospholipid metabolism	0.51	0.27, 0.96	0.0366	
1-Palmitoyl-2-arachidonoyl-GPC (16:0/20:4n6)	Phospholipid metabolism	0.55	0.30, 1.00	0.0486	
1-(1-Enyl-palmitoyl)-2-arachidonoyl-GPE (P-16:0/20:4)	Plasmalogen	0.45	0.24, 0.87	0.0181	
Glycocholenate sulfate	Secondary bile acid metabolism	0.46	0.23, 0.91	0.0255	
Lignoceroyl sphingomyelin (d18:1/24:0)	Sphingolipid metabolism	0.19	0.05, 0.63	0.0072	
Glycosyl-N-(2-hydroxynervonoyl)-sphingosine (d18:1/24:1(2OH))	Sphingolipid metabolism	0.51	0.27, 0.96	0.0361	
Behenoyl dihydrosphingomyelin (d18:0/22:0)	Sphingolipid metabolism	0.46	0.22, 0.95	0.0372	
4-Cholesten-3-one	Sterol	0.54	0.29, 0.98	0.0441	
**Peptides**					0.70
N-Acetylcarnosine	Dipeptide derivative	0.40	0.17, 0.91	0.0290	
**Xenobiotics**					0.40
Hippurate	Benzoate metabolism	1.69	1.01, 2.83	0.0449	
Eugenol sulfate	Food component/plant	0.46	0.24, 0.87	0.0170	
Tartarate	Food component/plant	0.52	0.28, 0.95	0.0330	
4-Allylphenol sulfate	Food component/plant	0.51	0.26, 0.99	0.0478	

[1]Conditional logistic regression models were used to estimate odds ratio and their 95% confidence intervals (CIs), with only matching factors adjusted in the model. The odds ratio is for one standard deviation increase in metabolite level. The table is sorted by chemical class, sub-pathway, and p value. The analysis is based on 33 cases and 33 controls.

Supplementary Table 3: Serum metabolites related to glioma according to median caffeine intake

Metabolite	Odds Ratio	95% CI	*P* value
Xanthine metabolism			
1-Methylurate	1.58	1.08, 2.30	0.017
Caffeine intake*: low	1.70	0.90, 3.24	0.59
Caffeine intake: high	1.49	0.88, 2.54	0.95
1-Methylxanthine	1.63	1.09, 2.46	0.018
Caffeine intake: low	1.62	0.89, 2.95	0.64
Caffeine intake: high	1.34	0.77. 2.35	0.91
Paraxanthine	1.52	1.05, 2.22	0.028
Caffeine intake: low	1.57	0.90, 2.77	0.61
Caffeine intake: high	1.29	0.76, 2.19	0.98
Theobromine	1.53	1.02, 2.28	0.038
Caffeine intake: low	1.39	0.76, 2.54	0.74
Caffeine intake: high	1.42	0.83, 2.41	0.97
5-Acetylamino-6-amino-3-methyluracil	1.55	1.02, 2.35	0.038
Caffeine intake: low	1.39	0.81, 2.40	0.69
Caffeine intake: high	1.33	0.73, 2.43	0.88
Theophylline	1.5	1.02, 2.22	0.041
Caffeine intake: low	1.57	0.86, 2.86	0.53
Caffeine intake: high	1.16	0.67, 1.99	0.96
7-Methylxanthine	1.47	1.01, 2.14	0.042
Caffeine intake: low	1.55	0.85, 2.83	0.73
Caffeine intake: high	1.36	0.81, 2.27	0.95

*10 individuals (7.8%) are missing caffeine intake data. Unconditional logistic regression model and age at enrollment was adjusted in the model. The odds ratio is for one-standard deviation increase in metabolite level.

Supplementary Table 4: Gene set analysis (GSA) for sub-pathway of serum metabolites in glioma: stratified by time from blood draw to glioma diagnosis (*P* < 0.05)

Sub-pathway	No. of contributing metabolites	*P* value from GSA
**Time from blood draw to glioma diagnosis: <9 years**		
Primary bile acid metabolism	8	0.034
**Time from blood draw to glioma diagnosis: ≥9 years**		
Urea cycle; arginine/proline metabolism	16	0.003
Glycolysis, gluconeogenesis, and pyruvate metabolism	5	0.013
Monoacylglycerol	14	0.017
Histidine metabolism	11	0.028
Food component/plant	32	0.036
Diacylglycerol	21	0.045
